# Funny waves in repolarisation and tachycardia in a patient suspected for Brugada syndrome

**DOI:** 10.1007/s12471-019-1291-9

**Published:** 2019-05-21

**Authors:** J. Shaffu, P. Goethals, L. Jordaens

**Affiliations:** 1 Kliniek Sint Jan, Brussels, Belgium; 2 Universitair Ziekenhuis Gent, Ghent, Belgium

A 37-year-old man was admitted after syncope with facial trauma. He had been examined for bradycardia 6 years earlier, had a known complete right bundle branch block (RBBB) without structural heart disease. Electrocardiography (ECG) findings (Fig. 1a) now showed a trifascicular block (RBBB, left posterior fascicular block and a first-degree atrioventricular block with a PR interval of 244 ms), and an ST elevation in V2 [1]. An epsilon wave in V2 [2] can be suspected. The left ventricular ejection fraction was 57%; the right ventricle had normal wall thickness, without dilatation, confirmed with magnetic resonance imaging. A monomorphic ventricular tachycardia was induced (Fig. 1b). He recognised this arrhythmia as his main complaint.

**Fig. 1:**
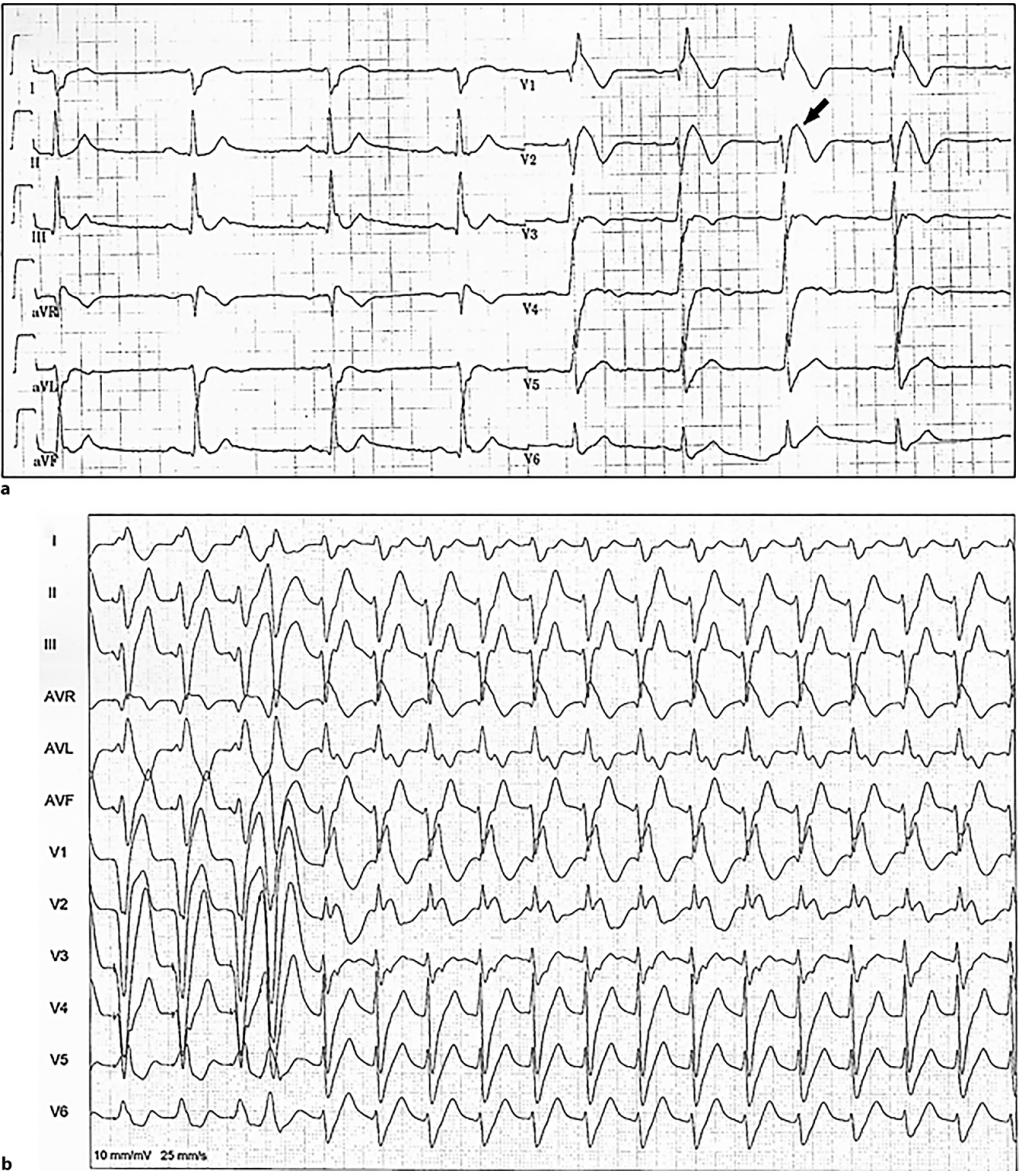
**a** Resting ECG with a complete RBBB and left posterior fascicular block. Observe the tall peaked wave in V1, superimposed on an ST-segment elevation of 4 mm in V1 and V2, with a distinct epsilon wave in V2 (*arrow*). **b** Induction of symptomatic, sustained wide complex tachycardia (cycle length 540 ms) with one ventricular extra-stimulus, on a drive train of 600 ms. The QRS width is 180 ms, with left axis and positive complexes in V1 and V2 (*ECG* electrocardiography, *RBBB* right bundle branch block)

Is this ECG compatible with Brugada syndrome? Are the anomalies in the right precordial ST segments a sign of another disease? Is the arrhythmia related to Brugada syndrome?

## Answer

You will find the answer elsewhere in this issue.
